# Human Tumor Cell Proliferation Evaluated Using Manganese-Enhanced MRI

**DOI:** 10.1371/journal.pone.0030572

**Published:** 2012-02-17

**Authors:** Rod D. Braun, David Bissig, Robert North, Kerry S. Vistisen, Bruce A. Berkowitz

**Affiliations:** 1 Department of Anatomy and Cell Biology, Wayne State University School of Medicine, Detroit, Michigan, United States of America; 2 Barbara Ann Karmanos Cancer Institute, Wayne State University, Detroit, Michigan, United States of America; 3 Kresge Eye Institute, Wayne State University, Detroit, Michigan, United States of America; University of Texas, M.D. Anderson Cancer Center, United States of America

## Abstract

**Background:**

Tumor cell proliferation can depend on calcium entry across the cell membrane. As a first step toward the development of a non-invasive test of the extent of tumor cell proliferation *in vivo*, we tested the hypothesis that tumor cell uptake of a calcium surrogate, Mn^2+^ [measured with manganese-enhanced MRI (MEMRI)], is linked to proliferation rate *in vitro*.

**Methodology/Principal Findings:**

Proliferation rates were determined *in vitro* in three different human tumor cell lines: C918 and OCM-1 human uveal melanomas and PC-3 prostate carcinoma. Cells growing at different average proliferation rates were exposed to 1 mM MnCl_2_ for one hour and then thoroughly washed. MEMRI R_1_ values (longitudinal relaxation rates), which have a positive linear relationship with Mn^2+^ concentration, were then determined from cell pellets. Cell cycle distributions were determined using propidium iodide staining and flow cytometry. All three lines showed Mn^2+^-induced increases in R_1_ compared to cells not exposed to Mn^2+^. C918 and PC-3 cells each showed a significant, positive correlation between MEMRI R_1_ values and proliferation rate (p≤0.005), while OCM-1 cells showed no significant correlation. Preliminary, general modeling of these positive relationships suggested that pellet R_1_ for the PC-3 cells, but not for the C918 cells, could be adequately described by simply accounting for changes in the distribution of the cell cycle-dependent subpopulations in the pellet.

**Conclusions/Significance:**

These data clearly demonstrate the tumor-cell dependent nature of the relationship between proliferation and calcium influx, and underscore the usefulness of MEMRI as a non-invasive method for investigating this link. MEMRI is applicable to study tumors *in vivo*, and the present results raise the possibility of evaluating proliferation parameters of some tumor types *in vivo* using MEMRI.

## Introduction

Uncontrolled cellular proliferation is the hallmark of cancer, and proliferation rate, i.e., the rate of tumor cell division, is linked to prognosis for several types of cancer [1], [2], [3], [4]. Currently the only method to spatially monitor local tumor cell proliferation *in vivo* is positron emission tomography (PET), which uses the accumulation of ^18^F-labeled 39-deoxy-39-fluorothymidine (^18^F-FLT), fluorodeoxyglucose (^18^F-FDG), or 2–11^C^ thymidine (11^C^TdR) as a proliferation marker [5]. While application of PET as a method of detecting proliferation *in vivo* remains promising, its spatial resolution is limited compared to other imaging modalities, such as MRI. Bading and Shields acknowledge that “an effective and clinically practical means for the imaging of cell proliferation is still an unrealized objective.”[5].

Cell proliferation is usually associated with an increase in cytoplasmic calcium ion, either from the extracellular space or from intracellular calcium stores [6], [7], [8]. Much of the extracellular Ca^2+^ enters the cell via calcium-permeable channels [6], [7], [8]. Indeed, tumor cell proliferation has been specifically linked to calcium ion channel activity in some, but not all, tumors [6], [7], [9], [10], [11], suggesting that calcium ion channel activity could be a useful surrogate marker of tumor cell proliferation. A powerful method for investigating calcium ion channel activity *in vivo* is monitoring the extent of tissue uptake of manganese ion, Mn^2+^, a Ca^2+^ analog [12], [13]. Manganese can enter cells via calcium ion channels, particularly through voltage-gated channels [12], [13], although other routes, including transferrin receptor-mediated or DMT1-dependent routes, may also contribute [14], [15]. Importantly, Mn^2+^ accumulates intracellularly due to a slow rate of efflux and acts as an MRI contrast agent by increasing the tissue longitudinal relaxation rate (R_1_ = 1/T_1_) in proportion to manganese concentration [16], [17]. Manganese-enhanced MRI (MEMRI) has been successfully used to functionally image brain [16], [18], [19], [20], [21] and retinal [22], [23], [24] activity, as well as the activity of other tissues [25]. These considerations suggest that MEMRI might be usefully applied to monitor tumor cell proliferation.

Free Mn^2+^ ion is known to accumulate in tumors *in vivo* [25], [26], [27], [28], [29]. Previously, using MEMRI in a nude rat model, we demonstrated significant Mn^2+^ uptake by C918 uveal melanoma xenografts relative to surrounding tissues and speculated that the tumor-specific Mn^2+^ uptake may have been a result of high proliferation rates within the tumor [30].

In this study, we test the hypothesis that tumor cell uptake of a calcium surrogate, Mn^2+^ (measured with MEMRI), is linked to tumor cell proliferation rate and will be a biomarker of proliferation in at least some tumors. Specifically, the growth of three human tumor cell lines was characterized *in vitro* and their proliferation rates were correlated to MEMRI R_1_ (1/T_1_) values.

## Materials and Methods

### Human Tumor Cell Lines

Three different human tumor cell lines were used in this study. The human uveal melanoma cell lines C918 and OCM-1 were used, because we had previously shown that C918 cells took up Mn^2+^ *in vitro* and *in vivo* [30], and we wished to further investigate this class of tumor. The OCM-1 cells were originally cultured from a human choroidal melanoma specimen in the 1980's [31], while the C918 cells were derived from a patient tumor in 1996 at the University of Iowa [32]. To extend the analysis of Mn^2+^ uptake to a type of cancer with a higher clinical incidence, we also investigated the human prostate carcinoma line PC-3, which was originally generated from a bone metastasis of a grade IV prostatic adenocarcinoma [33]. All cells were maintained in RPMI media +10% fetal bovine serum (FBS) + antibiotic under standard incubating conditions. Cells were seeded into 6-well plates or standard tissue culture flasks at a density of 20.8 cells/mm^2^.

### Measurement of Proliferation Rate of Human Tumor Cell Lines

To describe the growth of the tumor cells *in vitro*, cells were trypsinized, harvested, and counted on different days after seeding. The cell density was determined by dividing the cell number by the area of the well or flask. The number of cell divisions over that period of days, D, was calculated as:
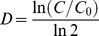
(1)where D = the number of cell divisions, C = tumor cell density (cells/mm^2^), and C_0_ = initial cell density, i.e., the seeding concentration (cells/mm^2^). These data were fit to a Weibull growth model [34] using nonlinear least-squares regression, while setting C_0_ = 20.8 cells/mm^2^ (GraphPad Prism, GraphPad Software, Inc., La Jolla, CA). The Weibull model was chosen after fit comparisons with other common growth models, including the Gompertz and sigmoid logistic models [35]. Model comparisons using Akaike's Information Criterion (AIC) method [36] showed that the Weibull model was the better model (AIC probabilities>99.5%) compared to either the Gompertz model or the sigmoid logistic model.

Since the Weibull model best described the growth of the cells, we used the corresponding equation to describe the number of cell divisions as a function of time:
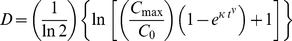
(2)where C_max_ = maximum cell density (cells/mm^2^), κ = the inverse of the time constant (1/days^ν^), and ν = dimensionless constant. The proliferation rate, dD/dt (divisions/day), is given by:
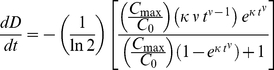
(3)


### MEMRI of Human Tumor Cell Pellets

Based on the tumor growth curves, cells were used for these experiments on different days after seeding. First 19.8 µl of stock MnCl_2_ solution (10 mg MnCl_2_•4H_2_O/ml of 0.9% saline; 50.5 mM MnCl_2_) was added to each ml of RPMI medium in the flask to reach a concentration of 1 mM MnCl_2_. The solution was left on the cells for one hour in the incubator. The cells were then rinsed with Hanks' balanced salt solution and trypsinized. After cell detachment was evident, the trypsin was quenched with RPMI media +10% FBS. The cells were centrifuged, and the resultant pellet was washed twice more in RPMI +10% FBS. The cells were counted and resuspended to a concentration of 1.5×10^6^ cells/100 µl. Two hundred µl of the suspension were placed in a 0.65 ml microcentrifuge tube, yielding a total of 3×10^6^ cells in the tube. The cells were pelleted by gravity for at least 20 minutes. At early time points, e.g., Days 2 or 3, it was sometimes necessary to use more than one flask to obtain 3×10^6^ cells. The microcentrifuge tubes were placed in a 7 T magnet (Bruker ClinScan , Billerica, MA), and the average longitudinal relaxation time (T_1_) for each pellet was determined using a partial saturation T_1_ approach as follows. Several spin-echo images (two 2.0 mm thick slices – one through pellets and one through supernatants – spaced 4 mm apart; matrix size 464×576, field of view 46×57 mm, TE 13 ms) were acquired at different repetition times in the following order (with number of acquisitions per TR in parentheses): TR 0.15 s (6), 3.50 s (1), 1.00 s (2), 1.90 s (1), 0.35 s (4), 2.70 s (1), 0.25 s (5), and 0.50 s (3). Images acquired with the same TR were averaged offline using ImageJ (http://rsbweb.nih.gov/ij/). Average signal intensity (SI, based on a circular region-of-interest encompassing most of the cells in each tube) from each sample varies as a function of TR according to a monoexponential function:
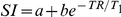
where a, b, and T_1_ are the fitted parameters. This function was fit to data using the Levenberg-Marquardt nonlinear least-squares algorithm in the minpack.lm library (v.1.1.1, by Timur V. Elzhov and Katharine M. Mullen) for R [37]. The R_1_ (1/T_1_) values (longitudinal relaxation rates) directly reflect manganese levels [16], [17].

Each pellet likely contained a heterogeneous cell population, e.g., cells in different phases of the cell cycle, and, potentially, correspondingly distinct values for R_1_, and this could confound our estimates of the pellet R_1_. As a first approximation, we assumed a fast-exchange limit wherein the exchange of water between heterogeneous compartments (including cell populations) is fast relative to the acquisition of the T_1_ data set. In this case, if the individual populations had different T_1_ values, that information would be blurred and would show up as a single exponential. To check this assumption, in a preliminary study using images from a typical run of nine pellets, we compared fits of monoexponential and biexponential functions to the intensity vs. TR data. When the data were fit to the monoexponential model, the fitted parameters had tight confidence intervals. In contrast, the biexponential fits resulted in parameter values with very large confidence intervals, indicative of non-unique parameter values (data not shown). Since these models are nested, i.e., one model is an extension of the other, an extra sum-of-squares F test was used to statistically compare the fits [36]. The F-test showed that the monoexponential fit was adequate to describe the data in every case and that there was no reason to invoke the more complex biexponential model (p>0.13, n = 9). These results are consistent with results from phantom experiments, which showed that T_1_ values have to differ by a factor of 2 to 3 to be reliably distinguished by a biexponential fit [38]. Therefore, all of the signal intensity data in this study were fit to the monoexponential equation noted above.

### Cell Cycle Analysis

For C918 cells, separate flasks were seeded at a concentration of 20.8 cells/mm^2^, and cells were harvested on different days after seeding. The washed cells were fixed in 70% ethanol by adding absolute ethanol dropwise to the cell suspension. The cells were stored at −20°C until flow cytometry could be performed on multiple samples. Three hours before flow cytometric analysis, the cells were washed three times in Hanks' balanced salt solution and were stained with a 50 µg/ml propidium iodide solution in the presence of 0.5 µg/ml RNase A. DNA content of each sample was determined by flow cytometry on a BD LSR II flow cytometer (BD Biosciences, San Jose, CA). The percentages of cells in G_0_/G_1_ phase, S phase, and G_2_/M phase were determined using ModFit LT software (Verity Software House, Topsham, ME). Flow cytometry was performed by the Microscopy, Imaging, and Cytometry Resources Core at The Karmanos Cancer Institute, Wayne State University.

For the PC-3 cells, a portion of the cells from the same flask that was used to perform the MEMRI experiments was processed for flow cytometry and analyzed in the same manner as described above.

To functionally describe the relationship between the percentage of cells in S phase and the proliferation rate, dD/dt, three different models were compared using Akaike's Information Criterion (AIC) method [36]: the sigmoid logistic model, the Weibull model, and a simple monoexponential model. The 3-parameter logistic model resulted in fits with lower sum-of-squares errors than the 3-parameter Weibull model for both cell lines. The AIC test revealed that the symmetric sigmoid logistic model was superior to the monoexponential model (AIC probability>70%). Therefore, the relationship is best described by:
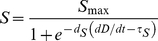
(4)where S = percentage of cells in S phase (%), dD/dt = proliferation rate (divisions/day), S_max_ = maximum percentage of cells in S phase (%), d_S_ = constant (days/division), and τ_S_ = value of dD/dt at which S is half its maximum value (divisions/day).

Forms of the same three functions were similarly compared to test their ability to adequately describe the relationship between the percentage of cells in G_0_/G_1_ phase and dD/dt. The 4-parameter logistic model resulted in fits with lower sum-of-squares errors than the 4-parameter Weibull model for both cell lines. Since the AIC test revealed that the symmetric sigmoid logistic model was the better model (probability>60%) compared to the monoexponential model, the sigmoid logistic model was used:
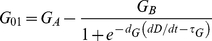
(5)where G_01_ = fraction of cells in G_0_/G_1_ phase (%), G_A_ = constant (%), G_B_ = constant (%), d_G_ = constant (days/division), and τ_G_ = constant (divisions/day). Note that G_A_ or G_B_ taken individually has no biological significance and could be >100, but G_A_-G_B_ is the minimum percentage of cells in G_0_/G_1_ phase (%).

The sum of all three cell fractions must be 100:
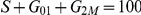
where G_2M_ = fraction of cells in G_2_/M phase (%). Solving this equation for G_2M_ yields:
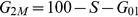
(6)


Substituting Equations 4 and 5 into Equation 6 yields the following expression for G_2M_:
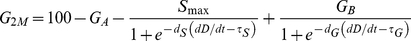
(7)


### Statistical Analysis

The fits of the cellular growth data to the logistic, Gompertz, and Weibull models were compared using Akaike's Information Criterion (AIC) method [36]. This test was used rather than an extra sum-of-squares F test, because the models are not interrelated, i.e., they are non-nested [36]. The AIC method was also used to compare the fits of the percentage of cells in S phase or G_0_/G_1_ phase versus proliferation rate data to the sigmoid logistic, Weibull, and exponential models.

Two-way ANOVA analysis was used to compare intracellular R_1_ values among the three cell lines with or without Mn^2+^ exposure. Tukey's HSD post-hoc comparison tests were used to check for differences between any two groups. Two-way ANOVA and Tukey's HSD were performed using R [37]. A p-value<0.05 was considered statistically significant.

Correlations between R_1_ values and proliferation rate were determined using linear regression analysis (GraphPad Software, Inc., La Jolla, CA). A regression p-value<0.05 was considered statistically significant.

The lack of fit of the weighted-average model to the experimental data was determined using the lack-of-fit ANOVA-based F-test [39], [40]. Using the sum of squares of the measurement and modeling errors, it tests the hypothesis that the lack of fit of the model curve is much greater than the measurement error [40]. A p-value<0.05 was considered statistically significant, indicating the model provided an incomplete description of the data.

## Results

### Tumor Cell Growth and Proliferation Rate

**Figure 1:**
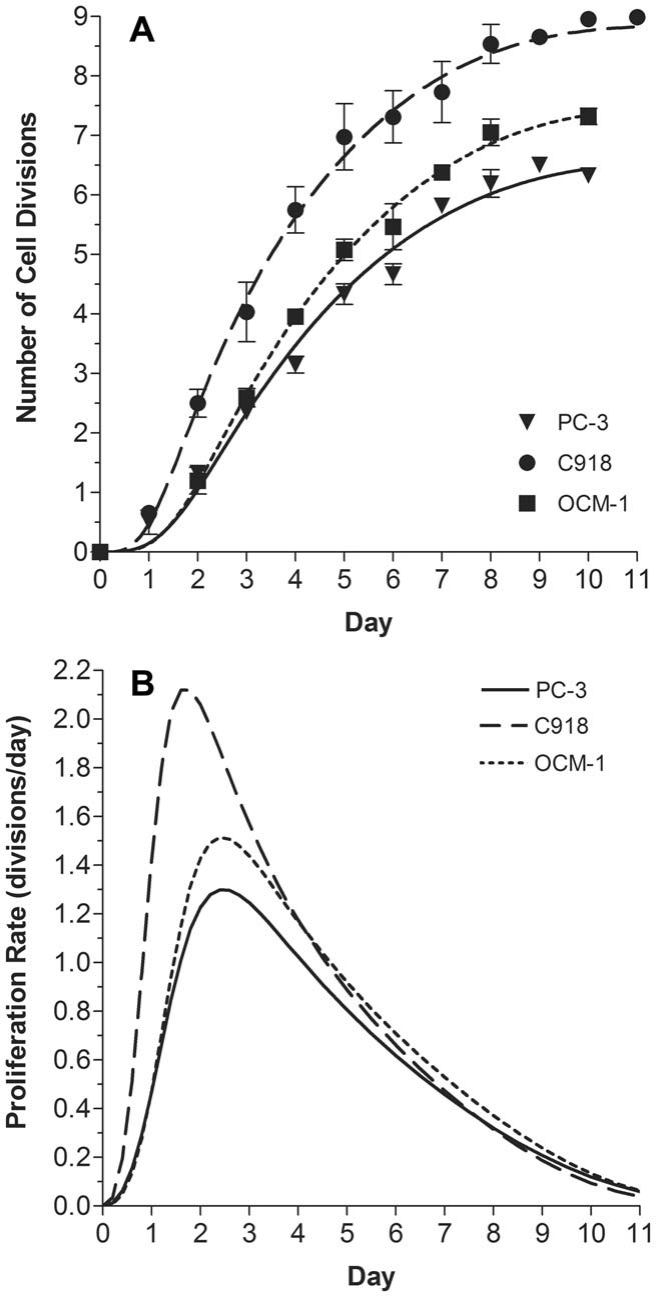
Tumor Cell Line Growth and Proliferation Rate. A) Growth of three human tumor cell lines, expressed as number of divisions after seeding at a concentration of 20.8 cells/mm^2^ on Day 0. PC-3 (▾, n = 84), C918 (•, n = 102), and OCM-1 (▪, n = 72) data were fit to the Weibull model (Equation 2). For clarity the mean ± SEM on each day are shown. Fitted parameters: PC-3: C_max_ = 1988, κ = −0.00115, ν = 3.30, r^2^ = 0.972; C918: C_max_ = 9636, κ = −0.000844, ν = 3.52, r^2^ = 0.969; OCM-1: C_max_ = 3748, κ = −0.000553, ν = 3.62, r^2^ = 0.969. B) Proliferation rates of the cell lines, as calculated from Equation 3: PC-3 (——), C918 (— —), and OCM-1 (----).

As shown in Figure 1, the growth data for all three cell lines were well described by the Weibull model (Equation 2), with all coefficients of determination equal to 0.97. Proliferation rates were calculated from the fitted parameters using Equation 3 (Figure 1B). C918 cells had the highest maximum proliferation rate of 2.1 divisions/day, which occurred 1.7 days after seeding. The OCM-1 cell line showed a maximum proliferation rate of 1.5 divisions/day at 2.4 days after seeding. The PC-3 cells grew the slowest with a maximum proliferation rate of 1.3 divisions/day at 2.5 days after seeding.

### Tumor Cell Mn^2+^ Uptake

**Figure 2:**
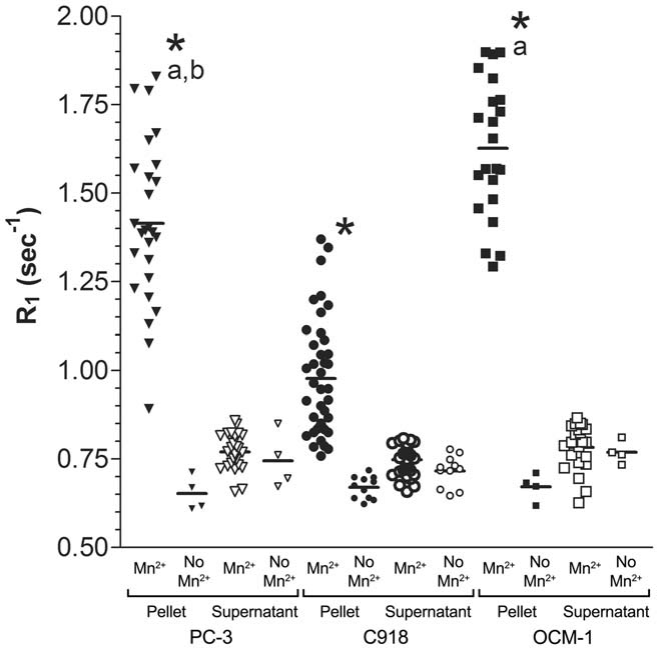
Comparison of Tumor Cell Pellet and Supernatant R_1_ Values. Average R_1_ values for tumor cell pellets (solid symbols) and supernatants (open symbols) after exposure to 1 mM MnCl_2_ or media without added Mn^2+^. Two-way ANOVA analysis of cell pellet R_1_ values revealed a significant impact of Mn^2+^ exposure (p<0.0001) and cell type (p<0.0001). Tukey's HSD test, Mn^2+^-exposed vs. no Mn^2+^: *p<0.0001. Tukey's HSD test, cell lines: a: p<0.0001 vs. C918 pellet, b: p = 0.001 vs. OCM-1 pellet. When no Mn^2+^ was added, there were no differences among the pellet R_1_ values (Tukey's HSD test, p = 1.000).

A 2×3 two-way ANOVA analysis revealed a significant impact of Mn^2+^ exposure (p<0.0001) and of cell type (p<0.0001) on the R_1_ value of the cell pellets, regardless of their proliferation rate (Figure 2). Post-hoc analysis demonstrated that, as expected, for each cell line, the R_1_ of cells exposed to Mn^2+^ for one hour and then rinsed was significantly greater than the R_1_ of corresponding cells not exposed to Mn^2+^ (p<0.0001, Tukey's HSD test, Figure 2). R_1_, and thus Mn^2+^ uptake, in the PC-3 and OCM-1 cell lines was greater than R_1_ in the C918 cells (p<0.0001). In addition, the R_1_ value of the PC-3 cells was significantly less than that of the OCM-1 cells (p = 0.001). When no Mn^2+^ was added, there were no differences among any of the pellet R_1_ values (Tukey's HSD test, p = 1.000). Supernatant R_1_ values, regardless of whether the cells were exposed to Mn^2+^ or not, ranged between 0.63 and 0.87 sec^−1^.

### Relationship between Tumor Cell Mn^2+^ Uptake and Proliferation Rate

**Figure 3:**
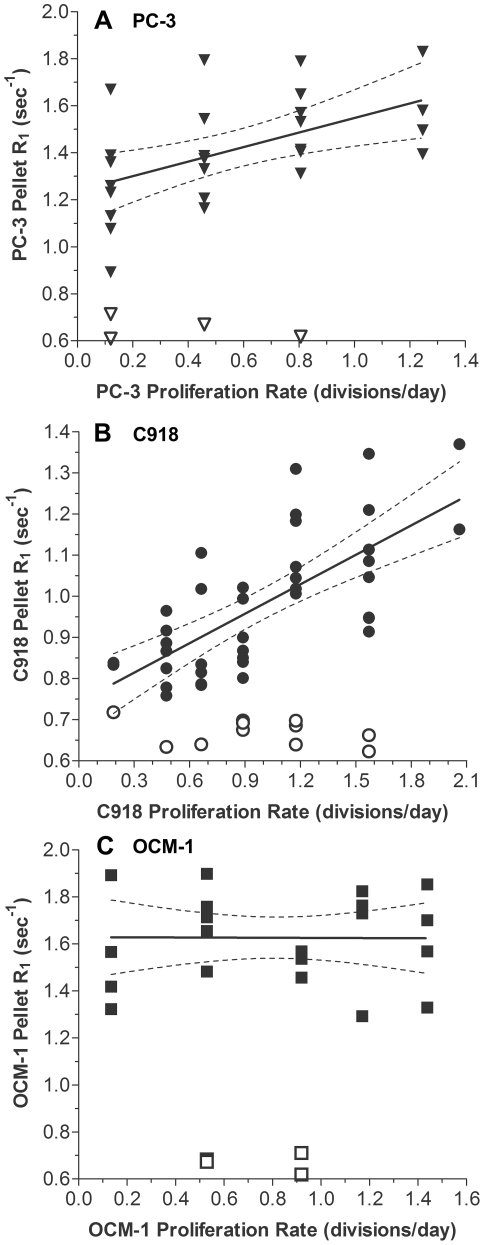
Relationships between Tumor Cell Pellet R_1_ and Proliferation Rate. Correlation between MEMRI R_1_ values in tumor cell pellets exposed to Mn^2+^ (solid symbols) and cellular proliferation rate as calculated from Figure 1B for A) PC-3 prostate carcinoma (▾), B) C918 uveal melanoma (•), and C) OCM-1 uveal melanoma (▪). Linear regressions: PC-3: R_1_ = 0.310(proliferation rate)+1.239; r^2^ = 0.284; n = 26, p = 0.0005. C918: R_1_ = 0.239(proliferation rate)+0.744; r^2^ = 0.502; n = 40, p<0.0001. OCM-1: R_1_ = -0.00294(proliferation rate)+1.63; r^2^ = 4.94×10^−5^; n = 22, p = 0.975. Open symbols represent R_1_ values of cell pellets in the absence of Mn^2+^. Note that the abscissas and ordinates have different scales in each panel.

The R_1_ values for pellets of cells following MnCl_2_ exposure are shown as a function of proliferation rate in Figure 3. In PC-3 prostate cancer cells, the Mn^2+^-enhanced R_1_ values for the cell pellets were positively correlated with proliferation rate (Figure 3A, r^2^ = 0.284, p = 0.005, n = 26), while the R_1_ values of cell pellets in the absence of Mn^2+^ were not correlated with proliferation rate (r^2^ = 0.146, p = 0.618, n = 4). C918 human uveal melanoma cells also took up Mn^2+^ (Figure 3B), and there was a significantly positive correlation between MEMRI R_1_ and proliferation rate (r^2^ = 0.502, p<0.0001, n = 40). Again, R_1_ values of cell pellets in the absence of Mn^2+^ were not correlated with proliferation rate (r^2^ = 0.111, p = 0.317, n = 11). The OCM-1 cells showed no correlation between Mn^2+^-dependent R_1_ and proliferation rate (Figure 3C, r^2^ = 4.94×10^−5^, p = 0.975, n = 22) or between R_1_ and proliferation rate in the absence of Mn^2+^ (r^2^ = 0.040, p = 0.799, n = 4).

### Phases of the Cell Cycle and Proliferation Rate

**Figure 4:**
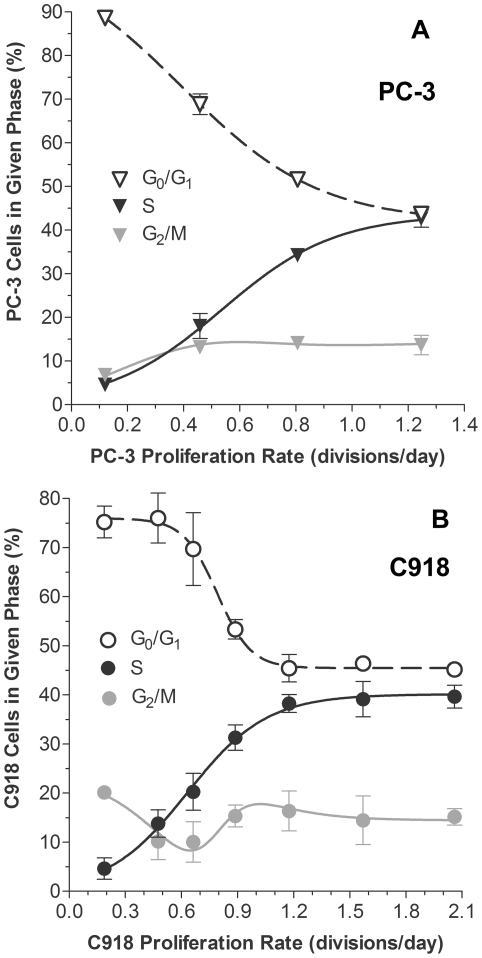
Relationships between Cell Cycle Fractions and Proliferation Rate. Percentage of cells in different phases of the cell cycle as a function of proliferation rate for PC-3 (A) and C918 (B) cells. S-phase data were fit to Equation 4. PC-3 (A): S_max_ = 43.6, d_s_ = 5.0, τ_s_ = 0.54 (r^2^ = 0.921, n = 29); C918 (B): S_max_ = 40.1, d_s_ = 4.7, τ_s_ = 0.63 (r^2^ = 0.920, n = 20). G_0_/G_1_ data points were fit to Equation 5. PC-3 (A): G_A_ = 104.2, G_B_ = 62.37, d_s_ = 4.05, τ_G_ = 0.392 (r^2^ = 0.967, n = 29); C918 (B): G_A_ = 75.9, G_B_ = 30.48, d_s_ = 11.13, τ_G_ = 0.791 (r^2^ = 0.848, n = 20). The curves describing the G_2_/M were calculated from Equation 7, using the above parameter values. For clarity the mean ± SEM on each day are shown (n = 3–10 values per point).

Since there was a positive correlation between MEMRI R_1_ values and proliferation rate in the PC-3 and C918 cell lines, cell cycle changes in those cell lines were also investigated. As expected, the fraction of cells in each of the phases of the cell cycle changed with proliferation rate (Figure 4). In both cell lines, the fraction of cells in S phase increased with proliferation rate, while the fraction of cells in G_0_/G_1_ decreased. The percentage of cells in S phase as a function of proliferation rate, dD/dt, was well described by Equation 4 for both the PC-3 cells (r^2^ = 0.921, n = 29, Figure 4A) and the C918 cells (r^2^ = 0.920, n = 20, Figure 4B). Similarly, Equation 5 adequately described the percentage of cells in the G_0_/G_1_ phase as a function of dD/dt for both the PC-3 cells (r^2^ = 0.967, n = 29, Figure 4A) and the C918 cells (r^2^ = 0.848, n = 20, Figure 4B). Equation 10 reasonably described the changes in the G_2_/M fraction of cells as well (Figure 4).

### Modeling the Relationship between Tumor Cell Mn^2+^ Uptake and Proliferation Rate

It is possible to derive a simple, general model to describe the positive correlation between Mn^2+^-induced changes in R_1_ and proliferation rate in the PC-3 and C918 cell lines (Figure 3). The cell pellets at each proliferation rate have at least three distinct subpopulations of cells in different phases of the cell cycle: G_0_/G_1_, S, and G_2_/M (Figure 4). If each subpopulation takes up a different amount of Mn^2+^, each subpopulation would have its own R_1_ value, and the average R_1_ of any mixed cell population would be the weighted average of these individual cell cycle-specific values. Thus, the overall R_1_ of the pellet is described by the following weighted average:
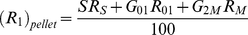
(8)where (R_1_)_pellet_ = R_1_ value of cell pellet (sec^−1^), R_S_ = R_1_ value of a cell in S phase (sec^−1^), R_01_ = R_1_ value of a cell in G_0_/G_1_ phase (sec^−1^), and R_M_ = R_1_ value of a cell in G_2_/M phase (sec^−1^). From Figure 4, it is known that the size of each subpopulation in the pellet changes with proliferation rate, and the parameters S, G_01_, and G_2M_ can be expressed as functions of proliferation rate. Substituting Equation 6 into Equation 8 and simplifying yields:
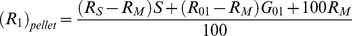
(9)Substituting Equations 4 and 5 into Equation 9 yields:
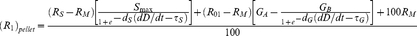
(10)The only unknowns in this equation are R_S_, R_01_, and R_M_. It is not known whether the amount of Mn^2+^ taken up by each subpopulation remains constant or changes with proliferation rate, i.e., R_S_, R_01_, and R_M_ are either constants or functions of dD/dt. Based on this reasoning, there are only two possible explanations for the positive correlation between the average cell pellet R_1_ and proliferation rate. Either it is attributable to proliferation rate-dependent changes in the fraction of cells in each subpopulation alone (R_S_, R_01_, and R_M_ are constant) or it is caused by proliferation rate-dependent changes in both the subpopulation distribution and in Mn^2+^ uptake of at least one of the subpopulations.

With the available data, it is possible to directly test the first possibility, i.e., that the three subpopulations demonstrate unique amounts of ion flux (R_01_, R_S_, and R_M_), but these amounts are not affected by proliferation rate. In other words, the correlation between R_1_ and proliferation is *solely* due to the relative size of each subpopulation. Application of this simple model will help guide future studies by indicating whether the second more complicated model needs to be further investigated.

The PC-3 and C918 cellular R_1_ values following MnCl_2_ exposure as a function of proliferation rate shown in Figure 3 were fit to Equation 10 (with R_S_, R_01_, and R_M_ as constants) using nonlinear least-squares regression (GraphPad Prism,GraphPad Software, Inc., La Jolla, CA). Cell line-specific constant parameters were obtained from the fits shown in Figure 4.

**Figure 5:**
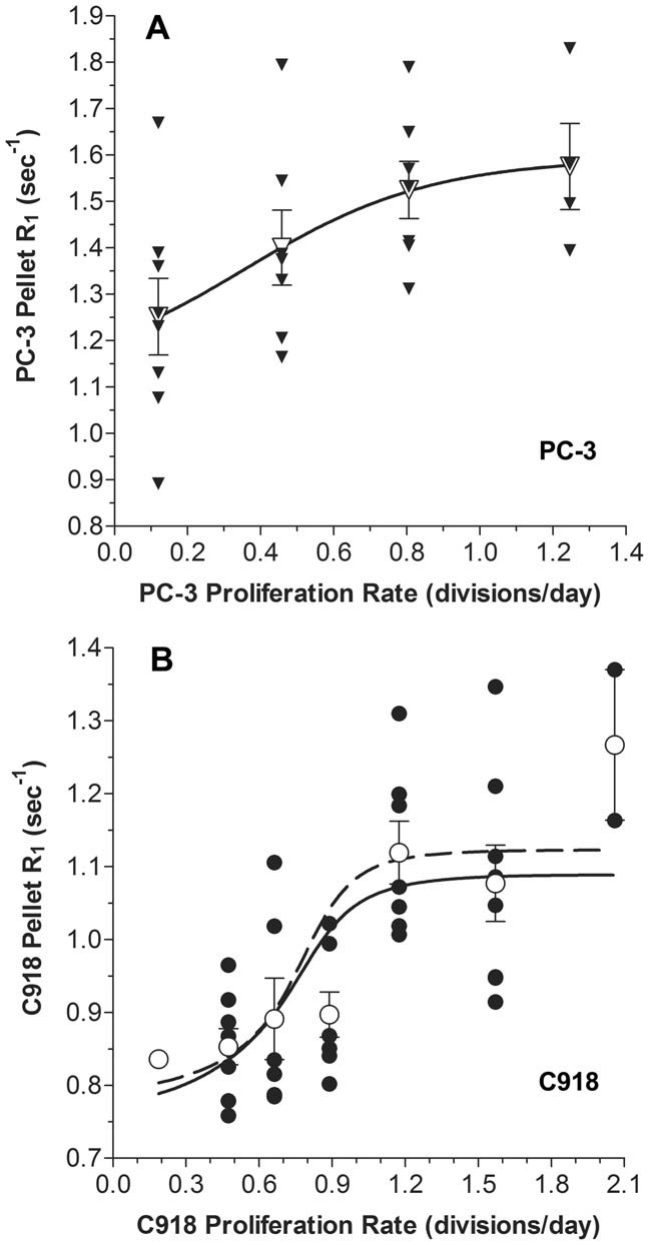
Weighted-Average Model Describing Cell Pellet MEMRI R_1_ Values as Function of Proliferation Rate. Solid symbols are the individual R_1_ values for PC-3 (A: ▾) or C918 cells (B: •) harvested on the same day of growth at the corresponding proliferation rate. Open symbols are the mean ± SEM of the R_1_ values for PC-3 (A: ▽) or C918 cells (B: ○). Solid lines show the fits of individual R_1_ values to Equation 10, using constants given in Figure 4. Fitted parameters: A) PC-3: R_01_ = 1.16 sec^−1^, R_S_ = 1.86 sec^−1^, and R_M_ = 2.02 sec^−1^; r^2^ = 0.304; n = 26. B) C918: R_01_ = 0.67 sec^−1^, R_S_ = 1.57 sec^−1^, and R_M_ = 1.07 sec^−1^; r^2^ = 0.436; n = 40. Dashed lines show the fits of the mean R_1_ values to Equation 10, using constants given in Figure 4. Fitted parameters: A) PC-3: R_01_ = 1.16 sec^−1^, R_S_ = 1.86 sec^−1^, and R_M_ = 2.02 sec^−1^; r^2^ = 1.000; n = 4. B) C918: R_01_ = 0.64 sec^−1^, R_S_ = 1.62 sec^−1^, and R_M_ = 1.26 sec^−1^; r^2^ = 0.722; n = 7. Note that the dashed line underlies the solid line in panel A.

For the PC-3 cells, the model fit of all 26 data points (Figure 5A, solid line, r^2^ = 0.304, n = 26) passes through the mean values (open symbols). A lack-of-fit ANOVA-based F-test [39], [40] revealed that the model adequately described the data (p = 0.978). In other words, given the variance in the PC-3 data and the quality of the present model fit, it is not possible for an alternative model to produce a significantly better fit. The best-fit values of R_01_, R_S_, and R_M_ with the 95% confidence intervals (95% CI) were 1.16 (95% CI: 0.82 to 1.51) sec^−1^, 1.86 (95% CI: 0.66 to 3.07) sec^−1^, and 2.02 (95% CI: −1.87 to 5.91) sec^−1^, respectively. Despite the wide confidence intervals (a consequence of uncertainty in R_M_), if the value for R_01_ is fixed within its 95% CI (between 0.82 and 1.51 sec^−1^), all solutions yield fits in which R_01_<R_S_. As an additional check that R_01_<R_S_, the four mean values were fit to the model (Figure 5A, dashed line). In this case, the fit was indistinguishable from the curve obtained when all of the individual points were fit to the model (solid line). The resulting fit yielded an r^2^ of 1.000 (n = 4) and the following parameter values: R_01_ = 1.16 (95% CI: 1.07–1.25), R_S_ = 1.86 (95% CI: 1.54–2.18), and R_M_ = 2.02 (95% CI: 0.98–3.05). From the confidence intervals, it is evident that R_01_ is always less than R_S_, but that R_M_ cannot be as reliably predicted. Based on these results, the correlation between PC-3 pellet R_1_ and proliferation rate is consistent with a change in the sizes of the cellular subpopulations during proliferation, without a change in their individual R_1_ values.

For the C918 cells, a fit of the 40 cell pellet R_1_ values following MnCl_2_ exposure to Equation 10 yielded the curve shown in Figure 5B (solid line, r^2^ = 0.436, n = 40). The best-fit values of R_01_, R_S_, and R_M_ with the 95% confidence intervals (95% CI) were 0.67 (95% CI: 0.48 to 0.86) sec^−1^, 1.57 (95% CI: 1.17 to 1.97) sec^−1^, and 1.07 (95% CI: −0.20 to 2.33) sec^−1^, respectively. Although, on visual inspection, the model does follow the general trend of the data, there are notable deviations. At low proliferation rates, the model predicts a more rapid change in pellet R_1_ than seen in the data, resulting in a large overestimate of R_1_ at a proliferation rate of 0.9 divisions/day (Figure 5B). Similarly, at high proliferation rates, the model predicts a plateau in the pellet R_1_ value, which is not evident in the experimental data. Consistent with these observations, a lack-of-fit ANOVA-based F-test [39], [40] revealed that the model does not adequately describe the data (p = 0.017). The assumption of constant R_1_ values for C918 cells in the different phases of the cell cycle appears questionable.

## Discussion

In this study, in two of three cancer cell lines, we found that MEMRI R_1_ values, which reflect cellular Mn^2+^ uptake, changed with tumor cell proliferation rate. These data underscore the usefulness and sensitivity of MEMRI to the known heterogeneous nature of different tumor cell proliferation rates. Although we and others have shown that tumor cells accumulate Mn^2+^ when exposed to MnCl_2_ and that Mn^2+^ changes the cellular MEMRI R_1_ values [25], [27], [28], [29], [30], this is the first time that the Mn^2+^-induced R_1_ changes have been correlated with changes in proliferation rate. In other studies, increased tumor Mn^2+^ uptake has been correlated with increased tumor neuroendocrine activity [27] or tumor cell Mn-superoxide dismutase (Mn-SOD) levels [29].

### Tumor Cell Mn^2+^ Uptake

Tumor cell Mn^2+^ uptake could occur by several different mechanisms, including expression and/or activity of Ca^2+^ channels, transferrin receptors, and divalent metal-ion transporter-1 (DMT1) channels [14], [15]. Both voltage-gated Ca^2+^ channels (VGCC) [13], [41], [42] and some TRP channels [43], [44], [45] are permeable to Mn^2+^, and the expression of these channels has been linked to tumor cell proliferation [6], [7], [10], [11], [46], [47], [48]. Although we cannot rule out a role for transferrin receptor-mediated or DMT1-dependent Mn^2+^ uptake, preliminary studies in our laboratory suggest that VGCCs, most likely T-type, are important regulators of Mn^2+^ uptake in the PC-3 and C918 cell lines. Therefore, we speculate that the difference in MEMRI R_1_ values, i.e., Mn^2+^ uptake, among the three cell lines, is related primarily to differences in the expression or activity of VGCCs.

The differences in Mn^2+^ uptake and cellular R_1_ values among the three cell lines (Figure 2) are most likely the result of different expression or activity of various calcium ion channels or other transport mechanisms responsible for Mn^2+^ entry into the cells. Interestingly, the C918 cells took up the least Mn^2+^, i.e., had the lowest pellet R_1_ values, even though they had the highest proliferation rates (Figure 1B). The OCM-1 cells, in which Mn^2+^ uptake was not correlated with proliferation rate, had the highest R_1_ values and an intermediate proliferation rate. These results demonstrate that the absolute level of Mn^2+^ uptake (R_1_ value) is not correlated with proliferation rate across cell types. In other words, a high R_1_ value does not mean that a particular cell line proliferates more rapidly than another cell line with a lower average R_1_. Rather, it is the change in the pellet R_1_ value for a specific cell line that is indicative of a change in cellular proliferation rate.

As expected, substantial leakage of Mn^2+^ out of the cells into the overlying supernatant did not occur, suggesting stable intracellular accumulation of manganese and adequate wash procedures. The lack of any significant Mn^2+^ leakage out of the cells after removal of the MnCl_2_ and washing of the cells implies a relatively slow rate of Mn^2+^ efflux out of the cells, similar to what has been reported in neuronal tissues [23], [49].

### Relationship between Tumor Cell Pellet MEMRI R_1_ and Proliferation Rate

Our initial hypothesis was based on reports that intracellular Ca^2+^ levels change during cellular proliferation and that these changes may, at least in part, be caused by an increase in the uptake of extracellular Ca^2+^ [6], [7], [8]. Since Mn^2+^ is a calcium surrogate, this uptake of Ca^2+^ might be detectable as a change in intracellular Mn^2+^ measured using MEMRI. Thus, we hypothesized a positive correlation between average MEMRI tumor cell pellet R_1_ value and cellular proliferation rate.

Two of the three tumor cell lines tested, PC-3 and C918, revealed such a positive correlation between average cell pellet MEMRI R_1_ and proliferation rate. No correlation was found for the OCM-1 uveal melanoma cell line. There are several possible, but not mutually exclusive, explanations for this result. First, it is possible that the proposed link between increased Ca^2+^ influx and proliferation is not universal to all tumor cell lines. For example, in the OCM-1 cells, the cytosolic Ca^2+^ change that helps drive proliferation may be dominated by release of intracellular Ca^2+^ stores, rather than uptake of extracellular Ca^2+^ [6], [7], [8]. Alternatively, extracellular Ca^2+^ may enter the OCM-1 cells through Mn^2+^-impermeable Ca^2+^ channels, so that the Ca^2+^ changes are not detected by MEMRI. Third, while a proliferation-related increase in Mn^2+^ uptake may occur through certain routes, the change in MEMRI R_1_ could be too small to be detected over the background of high baseline Mn^2+^ permeability through alternative proliferation-independent routes. This last possibility is consistent with the fact that the OCM-1 cells had the highest Mn^2+^ uptake (R_1_) of all three cell lines, regardless of the proliferation status (Figure 2). At this point, additional work is needed to identify the underlying reasons for a lack of a significant correlation in the OCM-1 cells. In any event, these data highlight the sensitivity of MEMRI to differential cell calcium handling in various tumor cell types.

As presented in the Results section, the simple weighted-average model (Equation 10) assumes that the changes in pellet R_1_ can be fully explained by changes in the relative distribution of cells in different phases of the cell cycle (each with a cell-cycle specific constant R_1_) as proliferation rate changes. For the PC-3 cells, the relationship between the average pellet R_1_ and proliferation rate was adequately described by this model (Figure 5A). The results of the modeling suggest that PC-3 cells in the G_0_/G_1_ phase of the cell cycle took up less Mn^2+^, i.e., had a lower R_1_ value, than cells in S phase. The dramatic increase in the fraction of cells in S phase during proliferation and concomitant decrease in the fraction of G_0_/G_1_ cells (Figure 4A) seems most likely responsible for the positive correlation between PC-3 pellet R_1_ and proliferation rate (Figure 2A).

For the C918 cells, the poorer fit of the model to the data (Figure 5B) suggests that the simple weighted-average model with constant cell cycle-specific R_1_ values is insufficient to completely describe the relationship between average cell pellet R_1_ and proliferation rate. This result suggests that the Mn^2+^ uptake and the R_1_ value of one or more of the cell subpopulations changed with proliferation rate, but more work is needed to investigate this possibility.

In summary, MEMRI is a useful non-invasive method for accurately measuring the link between tumor calcium channel activity and tumor proliferation *in vitro*. Future studies will investigate whether these proliferation-related changes in MEMRI R_1_ can be confirmed *in vivo*.
